# Homology modelling of protein-protein complexes: a simple method and its possibilities and limitations

**DOI:** 10.1186/1471-2105-9-427

**Published:** 2008-10-09

**Authors:** Guillaume Launay, Thomas Simonson

**Affiliations:** 1 Laboratoire de Biochimie (UMR CNRS 7654), Department of Biology, Ecole Polytechnique, 91128, Palaiseau, France

## Abstract

**Background:**

Structure-based computational methods are needed to help identify and characterize protein-protein complexes and their function. For individual proteins, the most successful technique is homology modelling. We investigate a simple extension of this technique to protein-protein complexes. We consider a large set of complexes of known structures, involving pairs of single-domain proteins. The complexes are compared with each other to establish their sequence and structural similarities and the relation between the two. Compared to earlier studies, a simpler dataset, a simpler structural alignment procedure, and an additional energy criterion are used. Next, we compare the Xray structures to models obtained by threading the native sequence onto other, homologous complexes. An elementary requirement for a successful energy function is to rank the native structure above any threaded structure. We use the DFIRE_*β *_energy function, whose quality and complexity are typical of the models used today. Finally, we compare near-native models to distinctly non-native models.

**Results:**

If weakly stable complexes are excluded (defined by a binding energy cutoff), as well as a few unusual complexes, a simple homology principle holds: complexes that share more than 35% sequence identity share similar structures and interaction modes; this principle was less clearcut in earlier studies. The energy function was then tested for its ability to identify experimental structures among sets of decoys, produced by a simple threading procedure. On average, the experimental structure is ranked above 92% of the alternate structures. Thus, discrimination of the native structure is good but not perfect. The discrimination of near-native structures is fair. Typically, a single, alternate, non-native binding mode exists that has a native-like energy. Some of the associated failures may correspond to genuine, alternate binding modes and/or native complexes that are artefacts of the crystal environment. In other cases, additional model filtering with more sophisticated tools is needed.

**Conclusion:**

The results suggest that the simple modelling procedure applied here could help identify and characterize protein-protein complexes. The next step is to apply it on a genomic scale.

## Background

Many cellular functions are mediated by protein-protein interactions [1-3]. An aim of modern genomics is to identify and characterize these interactions. With hundreds of genomes completely sequenced, computational methods that exploit sequence data are an attractive goal. Methods have been proposed to identify, classify, and validate putative interactions [4-8]. For example, amino acids that participate in a stable protein-protein interface tend to undergo correlated mutations during evolution, providing an interaction signature [4,9,10]; proteins that interact, physically or functionally, have an increased chance of being encoded by genes that are physically nearby, so that analyses of genome structure can also provide information on protein interactions [5,6].

Structure-based computational methods provide additional information, and are especially useful to characterize direct, physical interactions between proteins [11-19]. Docking algorithms are increasingly powerful and can provide a detailed description of the interaction [13,20-22]; however, they are too costly for routine large scale studies. A more attractive possibility is to perform homology modelling, and exploit the ever-growing structural databases [23] to model putative protein-protein complexes [16,18]. Homology modeling of a protein-protein complex requires that a structural similarity principle should hold: similar proteins should interact in a similar way. Previous studies show that this is true in many cases [24]. However, exceptions have also been found, where two homologous pairs of proteins interact in two very different ways [25]. In particular, a recent, systematic study by Aloy & Russell [24] found only a moderate correlation between sequence and structural similarity for protein-protein complexes.

Here, we examine the possibilities and limitations of a simple homology modelling procedure for protein-protein complexes. The method is closely related to earlier methods [18], but differs in many significant details. Ultimately, the method should allow us to predict whether a pair of proteins interact, given that a homologous pair forms a complex of known structure. We focus on the simplest type of protein-protein interactions: stable interactions between pairs of monodomain proteins. Domains are tightly-packed, globular structures. Their interactions can be considered as fairly representative of the interactions that take place between larger proteins [12,14,15]. Protein domains are usually thought to be structures that have been conserved in the course of evolution. The SCOP database [26], for example, provides a hierarchical classification of domains of known 3D structure according to their probable evolutionary relationships. Thus, domains are the logical starting point to attempt homology modelling.

As a first step, we examine the structural and sequence similarity among known domain-domain complexes. This question has been examined at length in the past [15,24]. Here, we use a slightly simpler dataset (binary complexes between single-domain proteins), a simpler method to align and superimpose pairs of complexes, and an additional energy criterion. A simple structural homology principle holds for most cases. Excluding complexes with weak association energies (which may be artefacts of the crystal environment), most complexes in the dataset that share over 35% sequence identity always share similar interaction modes and structures. Note that we are referring here to the sequence identity averaged over the whole complex, *not *just the interface region. This result is more clearcut than the earlier studies.

As a second step, we examine the performance of a simple homology modelling approach, which could be used for large-scale studies. It combines structure-based alignments with a very simple threading procedure and an empirical energy function. For a given domain-domain complex, several possible templates are considered, made up of homologous complexes. We compare the association energy of experimental complex to the energies of the modelled complexes. This test represents the most basic requirement for a successful energy function. With the simple, DFIRE_*β *_energy function [21], discrimination is good but not perfect for this relatively easy test.

Finally, we perform a more realistic test, comparing near-native structures to distinctly non-native models. Most near-native structures are ranked above most non-native models. Typically, however, a single alternate, non-native binding mode is found with a native-like energy. In some cases, this could correspond to a genuine binding mode; in other cases, such modes artefacts that would have to be filtered out using more sophisticated models.

Overall, it appears that our simple homology modelling, despite its limitations, can help identify and characterize protein-protein complexes, at least in a preliminary way. The method is computationally inexpensive and could be applied on a large scale. It can also be used in combination with other, purely sequence-based methods. In the following, we describe the Results. The next section is a Discussion. Computational Methods are described last.

## Results

### Datasets of protein-protein complexes

Three datasets of protein-protein complexes are used. Importantly, all the complexes are formed from pairs of single-domain proteins. Two of the datasets are from previous publications [15,18,27]. The first, "Keskin" dataset [15,27] includes 21686 pairs of domains, divided into 3799 groups of complexes with similar interaction modes. It will be used to judge the accuracy of our structural alignments. Indeed, the domains in this set were carefully aligned and grouped into clusters by Keskin et al [15]; therefore, they provide a benchmark to check that our own alignment procedure is reasonable. The second, much smaller, "Aloy" dataset [18] includes 35 complexes, divided into nine functional groups. This dataset was used by Aloy and Russell to test their own, earlier modelling procedure [18]. By applying our procedure to this dataset, we can directly compare our performance to theirs, and judge the accuracy of our procedure.

Finally, a third set of complexes was constructed here, larger than the Aloy set, based on the SCOP classification, and comprising only single domain protein complexes (unlike the Keskin set). Most members of this dataset are also found in the Keskin set. This set was constructed as follows. The starting point was the ASTRAL Compendium of SCOP domains [26,28], which is based on version 1.67 of SCOP and contains 65122 domains. From the Protein Data Bank [23], we collected all structures that included more than one chain. Cross-checking with the ASTRAL Compendium [26,28], we discarded structures that contained more than two SCOP domains or were absent from ASTRAL. We also required that the two domains be continuous and carried by different polypeptide chains, thus excluding complexes between two domains within the same protein. We were left with 4765 structures. To exclude complexes that are obviously non-biological, such as crystal contacts, we discarded structures that had fewer than ten contacts between their two domains. An interface contact was said to occur when one domain of the complex had a nonhydrogen atom less than 8 Å from a nonhydrogen atom of the other domain [29,30]. This left just 1509 complexes. Each one was checked with the program PQS [31]; only those for which PQS returned a dimeric status were retained. The 750 remaining complexes were partitioned into groups based on proximity in the SCOP classification. Specifically, two complexes A:B and A':B' were put in the same group if A and A' are part of the same SCOP superfamily (say, S_*A*_) *and *B and B' are part of the same SCOP superfamily (which can be different from S_*A*_). Groups containing three or fewer complexes were discarded, since they allow just a few threading models to be built (see below) and just a few discrimination tests to be done. At the end of this selection process, we were left with 743 domain-domain complexes, partitioned into 40 groups, with between 4 and 71 complexes per group. 667 complexes are homodimers; 66 are heterodimers. Each group will be labelled by its pair of SCOP superfamily identifiers. We will refer to each of the 40 groups as an "Interacting Superfamily Group", or ISG.

### Testing our alignments by classifying interaction modes

We first evaluate the accuracy of our structural alignments by comparing to the earlier, benchmark study by Keskin et al. [32]. The Keskin dataset contains 21686 complexes, clustered [27] according to the structural similarity of their binding modes [33]. 621 of the Keskin complexes are also part of our dataset. We performed a similar geometrical analysis of our own dataset, to identify the binding modes present in each Interacting Superfamily Group, or ISG (see Methods). All the complexes within each ISG (40 groups; 743 complexes in all) were compared to each other (see Methods), for a total of 9630 pairwise comparisons. The structural deviation is measured by an "Interaction rmsd", or Irmsd, which corresponds to the rms C_*α *_coordinate deviation between the smaller partners after superimposition of the larger partners; see Methods. The Irmsd was computed for each pair of complexes. A hierachical, average-linkage clustering was then performed, using the Irmsd as the distance metric. A maximum distance of 6 Å was allowed between any member of a cluster. With this procedure, each ISG yields a certain number of clusters, corresponding to distinct interaction modes between the two domains.

With this procedure, our clusters are in good agreement with the Keskin set (Table 1). This shows that our alignment method is reasonable. In particular, the use of a sequence alignment of regions that flank the MATRAS structural alignment (see Methods) does not cause difficulties. Most ISGs contain several interaction modes, most of which were also identified by Keskin et al. There are very few complexes from different ISGs that are clustered in the same interaction mode by Keskin et al. Only four clusters out of the 201 identified by Keskin et al. (Table 1) contain complexes from different ISGs (clusters 287, 653, 773, and 1133; data not shown).

**Table 1:** Binding modes within each Interacting Superfamily Group (ISG)

		number of modes		
interacting superfamilies	number of complexes^*a*^	this work	Keskin	overcounts^*b*^
c.67.1 c.67.1	71(65)	12	13	1
d.174.1 d.174.1	47(34)	2	2	1
c.37.1 c.37.1	41(32)	20	17	0^*c*^
b.1.1 b.1.1	39(18)	14	8	1
c.1.1 c.1.1	38	3	3	0
d.117.1 d.117.1	36	1	1	0
c.76.1 c.76.1	32(31)	2	1	0
c.2.1 c.2.1	26(25)	9	8	0
c.71.1 c.71.1	25(24)	6	5	0
d.169.1 d.169.1	21	5	5	0
b.60.1 b.60.1	21(19)	10	9	1
b.29.1 b.29.1	20(17)	7	9	0
e.3.1 e.3.1	18(13)	6	7	0
c.69.1 c.69.1	18(12)	14	10	0
d.17.4 d.17.4	17(11)	4	4	0
d.5.1 d.5.1	16	8	8	0
d.144.1 d.144.1	16(11)	12	7	0
c.68.1 c.68.1	16(12)	5	4	0
a.39.1 a.39.1	16(14)	7	6	0
e.7.1 e.7.1	15	2	2	0
a.133.1 a.133.1	15(12)	10	7	2
b.47.1 g.3.11	17	1	1	0
a.118.6 a.102.4	15(7)	1	1	0
b.47.1 b.47.1	13(11)	12	9	1
c.61.1 c.61.1	12	5	5	0
d.92.1 d.92.1	11	5	5	0
d.32.1 d.32.1	11(9)	3	3	0
c.94.1 c.94.1	11(7)	4	4	0
c.1.10 c.1.10	11(9)	5	4	0
c.52.1 c.52.1	10	6	5	1
d.1.1 d.1.1	9(5)	3	2	0
c.47.1 c.47.1	9(4)	6	4	0
a.1.1 a.1.1	9(8)	4	3	0
d.9.1 d.9.1	8(6)	3	3	0
b.6.1 b.6.1	8	1	1	0
a.123.1 a.123.1	8(4)	3	2	1
c.26.1 c.26.1	5(3)	4	3	0
c.66.1 c.66.1	4(2)	3	2	0
d.2.1 d.2.1	4	3	3	0
c.1.8 c.1.8	4	2	2	0
Total	743(621)	206	195	9

^*a*^In parentheses, the number of complexes that are part of the Keskin dataset [15]. ^*b*^Modes found in our study but not in the Keskin study. ^*c*^When a complex is not found in the Keskin set, and it is the only representative of its interaction mode, the mode is not considered an overcount. In the c.37.1/c.37.1 case, for example, we find 20 modes, but 3 are represented by a single complex each, not found in the Keskin dataset: there are no overcounts. ^*d*^In a few cases (eg, c.67.1 c.67.1), our dataset misses some of the Keskin modes.

Compared to the Keskin interaction modes, our analysis returned nine additional modes (Table 1). Most of these modes should probably be considered false positives, or "overcounts" of our mode counting. They correspond to structural similarities between interaction modes that were underestimated by our comparison method. Note that a few are genuine, since our dataset includes some additional complexes that were not part of the Keskin study.

Both our own and the Keskin analyses frequently identify very diverse modes of interaction within the same SCOP superfamily. In fact, this diversity is misleading. We will see below that the different interaction modes are almost always associated either with sets of complexes having a low mutual sequence identity, or with small, weakly-stable interfaces that are probably non-biological, induced by a given crystal environment.

### Relation between sequence and structure similarity

Homology modelling of protein-protein complexes is only viable if similar sequences lead to similar structures. To understand more clearly the relation between sequence and structural homology, we consider our 743 complexes, grouped into 40 ISGs. We begin by identifying complexes whose interfaces are sufficiently large and energetically stable. Specifically, we identify complexes that have a buried surface of at least 600 Å^2 ^and a DFIRE_*β *_interaction energy of -10 kcal/mol or better. Following earlier analyses of protein-protein complexes [29,30], we assume that complexes with such large and stable interfaces are likely to be biologically meaningful complexes, whereas the other complexes are much more likely to be artefacts of the crystal environment. The energy cutoff is a small but partly arbitrary value; see below. Of the 743 complexes, 30 are eliminated by their small surface areas. Another 233 are eliminated by the energy cutoff. We are left with 480 "large and stable" complexes.

**Figure 1:**
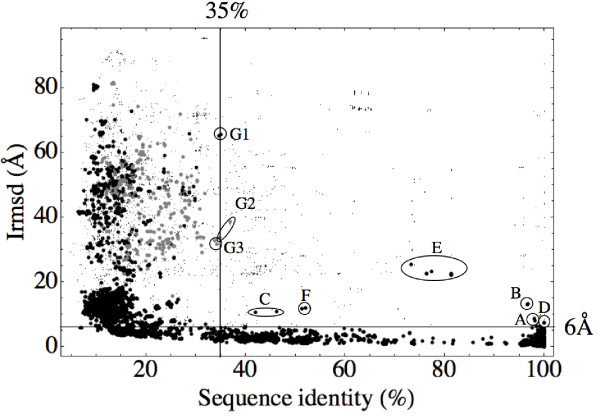
**The relationship between sequence and structural similarity**. 743 complexes from 40 interacting superfamily groups (ISGs) were analyzed. All pairs within each ISG were compared, for a total of 9630 pairwise comparisons. Small points correspond to comparisons involving at least one complex with either a small interface (buried area < 600 Å^2^) or a weak association energy (*E*_*int *_> -10 kcal/mol; see text). Points labelled A-G are discussed in the text. The horizontal line corresponds to Irmsd = 6 Å; the vertical line corresponds to a 35% sequence identity. Gray points correspond to comparisons where the MATRAS structural alignment provided fewer than 80% of the equivalent residues used for the Irmsd calculation. All the gray points lie below the 35% similarity threshold.

From each pairwise comparison, we obtain a sequence identity and a structural similarity score, as described in Methods. The sequence identity is given by the optimal alignment (structural, plus flanking sequence alignment if required; see Methods). The structural similarity is measured by the pairwise Irmsd (computed by first superimposing the larger members of a complex, say A_*i *_and A_*j*_, then comparing all equivalent C_*α*_'s of the smaller members, say B_*i *_and B_*j*_; see Methods). The Irmsd values are plotted vs. the corresponding sequence identities in Fig. 1 for the whole dataset (9630 points). In Fig. 1, each large dot corresponds to a comparison between two of the "large and stable" complexes. Each small dot corresponds to a comparison where one or (usually) both of the complexes being compared have small and/or less stable interfaces. Large, gray dots correspond to comparisons where the structural alignment with MATRAS provided fewer than 80% of the equivalent residues used for the Irmsd calculation; the rest of the equivalent residues are provided by a sequence alignment (see Methods). All the gray points lie in the lefthand part of the plot, below a 35% sequence identity. Thus, our use of a flanking sequence alignment for some complexes does not affect the qualitative results. We can now comment on our choice of energy cutoff: -10 kcal/mol is in fact the largest (least negative) value that gives a clean separation between the large and small dots in Figure 1.

In a previous study, Aloy and Russell extensively studied the relationship between the sequence and structural similarities of numerous protein complexes; see Fig. 1 in reference [24]. In contrast with their approach, which used a simplified geometric representation to measure structural deviations (four sites per subunit), we compute the Irmsd between pairs of complexes using an atomic level of detail. We also apply an energy cutoff that yields additional insights, and we use a simpler, more homogeneous dataset (binary complexes between single-domain proteins).

With these modifications, the sequence/structure homology principle is more clearcut. Indeed, considering only the large dots (black or gray), we see that when two complexes share at least 35% sequence identity between their subunits, they almost always have a similar mode of interaction. Note that the sequence identity is averaged here over the whole complex, not just the interface region. At this level of sequence identity, almost all of the measured Irmsd's are below 6 Å (Fig. 1). Thus, in the vast majority of cases, sequence homology implies structural homology. This is a basic requirement to allow homology modelling of protein-protein complexes. Below 35% of sequence identity, the interaction modes often differ.

In contrast, the small dots are widely distributed throughout Fig. 1. One group of points, for example, forms a band at around 100% sequence identity, extending up to Irmsd values of 80 Å. These points presumably arise from different packing modes in different crystal forms. A detailed analysis of crystal packing effects is beyond the scope of this paper; see [29,30].

In some cases, two "large and stable" complexes have subunits that share more than 35% sequence identity and yet they do present strongly dissimilar interaction modes (Irmsd above 6 Å). In Fig. 1, such complexes form nine groups, identified by the letters A-G. Group A involves two structures of the endonuclease EcoRV, which is a homodimer, either bound to DNA (1BSU) or unbound (1RVE). Although the whole enzyme is considered to form a single domain by SCOP (c.52.1 superfamily), the structure is actually made of two distinct, globular regions. Upon binding to the DNA, there is a relative motion of these two regions, which leads to a fairly high Irmsd between the two structures (7.8 Å), despite their nearly identical sequences (98.3% identity). Group B corresponds to another two endonucleases, with the same structural change upon DNA binding. Group C is similar. One complex has a large overall Irmsd value with respect to two others; if only the regions close to the interface are compared, the structural deviations are very small (<2 Å). The large Irmsd values are thus produced by a compact, globular region far from the interface which could be considered as a separate domain [34]. All these cases can be considered artefacts of the SCOP domain definitions.

Group D involves the complex 1A64, a member of the Immunoglobulin-like ISG. The subunits of this complex are are artificially engineered mutants of the variable antibody domains. The mutants were specifically selected to produce different interaction modes [35]. Group E corresponds to Ribonuclease A structures. These homodimeric structures are known to provide remarkable examples of domain swapping [36,37]. This leads to geometrically different interfaces, despite identical or very similar sequences. Such phenomena are relatively rare among protein dimers. Group F corresponds to a dimeric snake toxin protein [38], compared to a complex of a homologous toxin with an inhibitor. The inhibitor is expressed in the snake to modulate the activity of the toxin [39]. Remarkably, the inhibitor is strongly homologous to the toxin (~50% identity), and modulates its action by forming a heterodimer with one of the toxin subunits. The mode of interaction between the toxin and the inhibitor differs from the toxin homodimerization mode. This is an unusual case where a new biological interface has evolved that violates the homology principle (similar sequences but different interaction modes).

Another region of interest is the one between 33 and 37% sequence identity. Here, there are three small groups with large Irmsd values: G1, G2, G3. Group G3 is another case where structural deviations arise from a domain swapping event (complexes 1E8A, 1HT9, 1KSO). Group G1 arises from three complexes (1DEK, 1KO5, 1KO8), two of which (1KO5, 1KO8) are very similar (Irmsd of 1.5 Å). Comparing 1DEK and the other two, the interaction modes are very different, despite a 35% sequence identity. However, the interfaces are not very large (~2000 Å^2^) and the DFIRE_*β *_association energies are about -11 kcal/mol, barely below our -10 kcal/mol threshold. Finally, Group G2 contains two complexes (1G64 and 1HOP) with different interaction modes despite a 35% sequence identity, large buried surfaces, and large association energies (<-20 kcal/mol). Thus, the region between 35 and 37% sequence identity is a limiting region, where the structural homology principle begins to be violated. Notice that the precise location of this threshold region (~35%) is a result of our precise method of sequence and structural comparison, and the precise energy cutoff employed; slightly different methods would lead to a slightly different threshold.

Overall, structural homology holds in the vast majority of cases when the sequence identity is above 35% and the association energy is sufficiently large.

### Recognition of native interfaces: comparing the experimental structure to threading models

The structural homology principle observed above (in agreement with earlier work [24]) is a necessary but not a sufficient condition for homology modelling of protein-protein complexes. We turn now to the problem of the energy function. We first perform a very simple test, both with DFIRE_*β *_and with a simpler, residue-based energy function [40]. Their discrimination power was evaluated by their ability to assign a lower association energy *E*_*int *_to the experimental, Xray structure, compared to alternate structures obtained by threading the sequence onto other, homologous dimers. This discrimination, again, is a basic, necessary condition for a successful modelling procedure. 17 groups of homologous dimers were used, for a total of 123 complexes. Eight of these groups, containing 88 complexes, came from our own, ISG dataset. Nine groups, containing 35 complexes, came from the work of Aloy and Russell [18], who performed the same discrimination test.

The DFIRE_*β *_function has a 92% success rate in the discrimination tests (1035 successes out of 1124 tests). The number of successful and failed tests are given in Table 2 for each superfamily group. Each sequence within a group is threaded onto every other structure in the group (see Methods). Success means that a positive *S*_thread _score is obtained when an experimental structure is compared to a threaded model (see Methods). The residue-based energy function has a respectable, but poorer discrimination rate of 66%. Among the structures used by Aloy and Russell, those from the peptidase and squash trypsin groups were the hardest to discriminate. This is in fair agreement with the results they reported [18], using a different energy function and a different measure of structural similarity.

**Table 2:** Discriminating experimental complexes from threaded models

Superfamily identifiers	Superfamily names	sequence Id ranges (%)	number of complexes	number of tests	number of successes^*a*^	
					DFIRE_*β*_	Launay
a.133.1	Phospholipase A2	33–99	11	110	77	62
d.17.4	NTF2-like	11–98	13	156	140	92
b.29.1	lectins	33–99	15	210	205	148
c.61.1	Ribosyltransferase	15–99	10	90	80	65
e.3.1	*β *lactamase-like	9–100	11	110	107	59
d.5.1	RNase A-like	25–94	6	30	26	17
c.2.1	Rossmann-fold	11–43	14	182	174	152
a.118.6 a.102.4	Prenylyltransferase	94–99	8	56	56	27
	RhoGDI/Ras	54–94/73–100	4	12	11	6
	FGF receptor/FGF	20–35/22–62	3	6	6	6
	Trypsin/inhibitor	30–100/10–100	12	132	127	87
	Trypsin/inhibitor	65–83/56–71	3	6	4	1
	Peptidase M10/TIMP	42–98/50–100	3	6	4	6
	Trypsin/inhibitor	14/98	2	2	2	1
	Trypsin/inhibitor	39/42	2	2	2	0
	Collicin, Pyocin/HNH	39/42	2	2	2	1
	Elongation factor/EF-TS	20–57/6–22	4	12	12	7
		**Total**	123	1124	1035	737

^*a*^The number of tests giving a positive S_*thread *_score. Both the 6-class, residue-based and the DFIRE_*β*_, atom-based were used for scoring.

36 complexes out of the 123 were not fully discriminated: their sequences had a better energy for at least one of the models than for their Xray structure (negative *S*_thread_). For most of these, either the model was very similar to the Xray structure (Irmsd values of 2 Å or less), or the Xray structure had a weak association energy (suggesting that it may not be a biological complex). We consider each case in detail.

In the b.29.1 group (15 complexes), there are four complexes not fully discriminated. For two, the Xray complex has a structure very similar to the model complex that leads, after threading, to a better association score (Irmsd values of 1–2 Å), so that these are very mild failures. The other two complexes both have weak DFIRE_*β *_association energies (-5 and -6 kcal/mol). In the c.61.1 group, four complexes are not discriminated. In each case, the Xray complex has a structure very similar to the model complex that outscores it (Irmsd values of 1.5 Å or less). The same is true for the d.17.4 group (seven complexes not discriminated, outscored by models very similar to them; Irmsd values of 1 Å or less). In the d.5.1 group, three complexes are not discriminated. One (1H8X) is a domain-swapped dimer (see above). The other two (1DYT and 2RNF) are enzyme homodimers where the monomer is functional and there is no indication in the literature of a functional dimer. In the e.3.1 group, there are three real failures, two of which correspond to complexes with weak association energies. In the c.2.1 group, there are 26 complexes (Table 1). Only 14 were used in the discrimination tests (Table 2), as several redundant complexes were excluded (100% sequence identity with other complexes in the group and very similar structures; for example 1fk8 and 1fjh, 1keu and 1ker). Five of the 14 complexes are not discriminated. Four of them are very similar to each other (Irmsd values of ~2 Å) so that, in fact, there are only two real failures in this group. For the two cases, the sequence identity between the Xray complex and the relevant model complex is around 25%.

Finally, in the a.133.1 group, there are 10 complexes not fully discriminated. For one (1AOK), the model that outscores it (1JLT) is very similar to the Xray complex (Irmsd = 1 Å). Of the other nine, eight have weak association energies, with DFIRE_*β *_scores between -3 and -8 kcal/mol. All eight prefer the same model, 1JLT. The 1JLT template provides the threaded sequences with a large interface of 3100 Å^2 ^and numerous interface contacts (50 interacting residues per subunit). We speculate that the eight Xray complexes could be artefacts of the crystal environment. For these complexes, 1JLT could represent a true biological interaction mode. The other failure (1PP2) corresponds to a large, stable interface; the sequence identity with the preferred model (1JLT, again) is about 50%.

Overall, if we include near successes, exclude very weak complexes, and take into account mutually similar, redundant structures, this first, basic, discrimination test has just 20 real failures.

### Recognition of native interfaces: comparing near-native and distinctly non-native models

**Figure 2:**
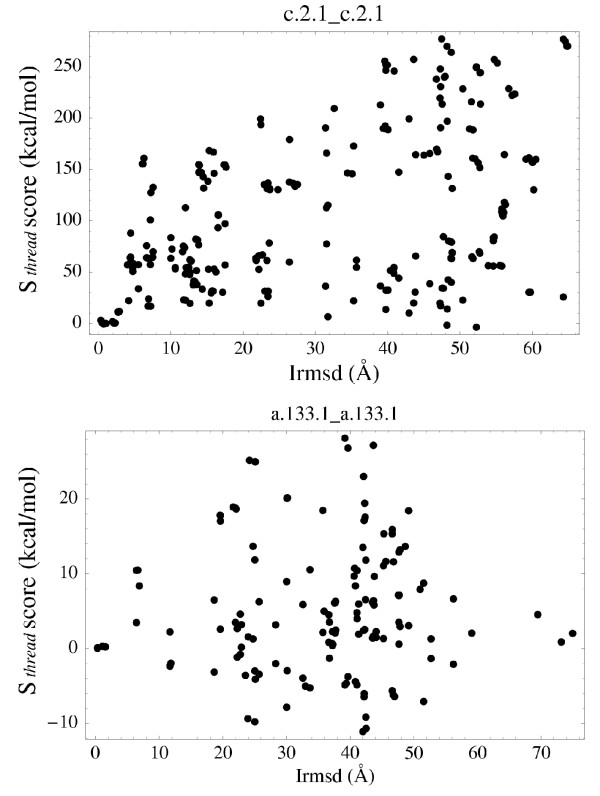
**S_*thread *_vs. Irmsd relative to the Xray structure**. Representation of all the modelled complexes within the c.2.1 (top) and a.133.1 (bottom) groups.

In a practical modelling situation, one does not know the experimental structure; rather, one must distinguish between models that are close to the real structure ("near-native" models) and others that deviate significantly from the true structure ("non-native" models). It is therefore of interest to examine the variation of the association energy as a function of the deviation Irmsd from the native structure. Ideally, a smooth increase should be observed. To illustrate the possibilities and limitations of the present modelling procedure, we focus on two ISGs in particular: c.2.1 and a.133.1. These groups are fairly representative of the full set of discrimination tests (Table 2). Fig. 2 shows the complete set of threading events for each group (650 and 210 events, respectively), relating each *S*_thread _score to the structural similarity (Irmsd) between the Xray structure and the corresponding model. The dataset includes both "non-native" and "near-native" tests; a "near-native" model occurs when the native sequence is threaded onto another complex that happens to be structurally homologous. The energy "surfaces" thus constructed appear complex, with no simple relation between *S*_thread _and Irmsd. We do not observe a smooth increase with Irmsd. A more detailed analysis shows, however, that the model does display a distinct, albeit imperfect correlation between energy and structure.

**Figure 3:**
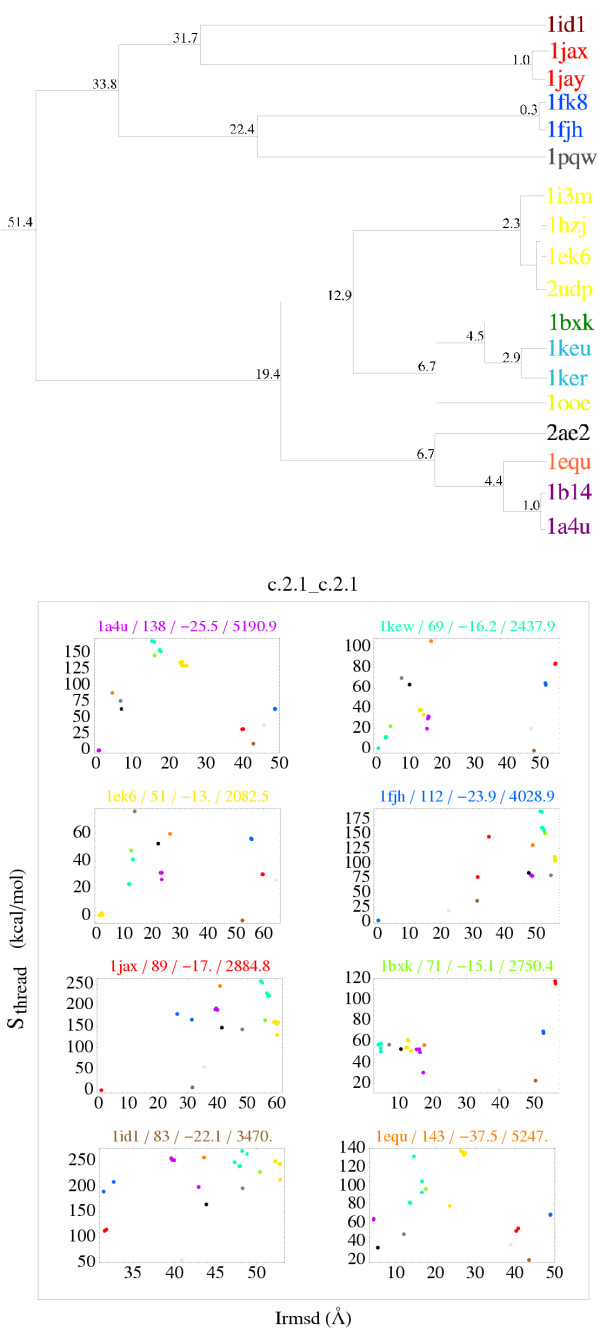
**c.2.1 group: representation of binding modes**. Above, tree representation of binding modes. Nodes are labelled with the mean Irmsd (Å) between leaves. Below, S_*thread *_vs. Irmsd relative to the Xray structure. Each panel corresponds to an Xray complex and is labelled with its PDB code, number of residue-residue contacts, *E*_*int *_(kcal/mol), and buried surface area (Å^2^). Each point corresponds to the threading onto a particular template; the color identifies the template; the point representing the Xray structure (zero S_*thread *_zero Irmsd) is not shown.

Indeed, Fig. 3 gives a more detailed view of the c.2.1 data. Several distinct binding modes were identified above (Table 1). Using a somewhat finer clustering here, we distinguish 11 modes (instead of nine in Table 1). These are shown in the upper part of Fig. 3. Complexes are colored according to their binding mode; eleven colors can be seen. For simplicity, only 18 complexes are shown in the Figure (of the 26 in this group; some very similar structures are left out of the Figure). In the lower part of Fig. 3, each panel corresponds to one Xray complex and one binding mode; the Xray complex is compared to all possible threading models. Only eight of the eleven binding modes are shown. With this detailed representation, the few, very mild discrimination failures within this group can be seen individually (points with a negative *S*_thread_; *e.g*., there are two failures in the 1EK6 and 1KEW panels, which correspond to the yellow and green binding modes). Except for a few points, *S*_thread _does tend to increase with Irmsd. This indicates a correlated energy surface. In the 1ID1 panel, for example, *S*_thread _increases rapidly then remains roughly constant for 40 ≤ Irmsd < 60 Å. In the 1JAX and 1EK6 panels, the situation is similar, except for one brown point. In all panels, any points with low energies but large Irmsds are associated with one or all of the red, blue, and especially brown binding modes. These binding modes are mutually similar; see tree in upper panel of Fig. 3. Clearly, this group of binding modes represents a strong competitor for several other, experimental modes. It may be that this group represents a genuine, alternate mode that could be observed under the right experimental conditions; it may also be that some of the other, experimental modes are artefacts of the crystal environment. Even if the low energy assigned to this group of modes is a pure artefact of the model, we note that our procedure has allowed most other modes to be correctly positioned in the high energy region. The few, low energy, competing structures (red, brown, and blue) all correspond to a single, competing binding mode that could possibly be discriminated by further, more sophisticated energy calculations such as all-atom models.

**Figure 4:**
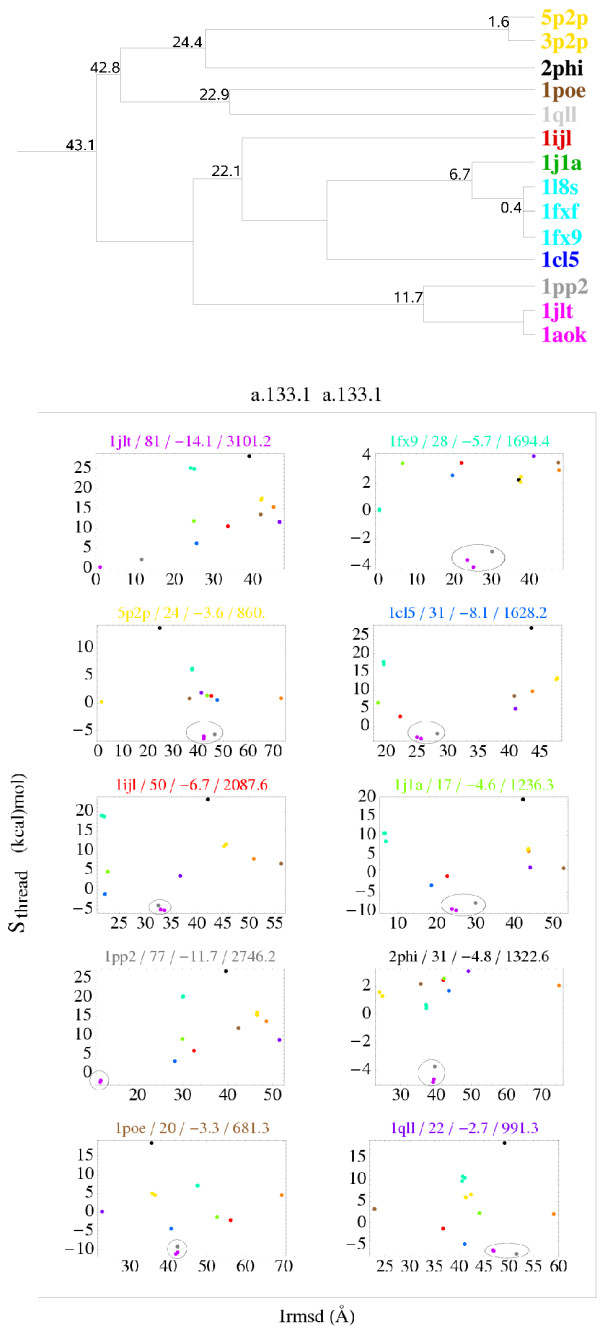
**a.133.1 group: representation of binding modes**. Same view as in Fig. 3. The largest discrimination failures in each panel are circled. They all correspond to sequences threaded onto the same model structures: 1JLT, 1AOK, and 1PP2, which have especially large interfaces (see text and panel headers).

Fig. 4 shows the a.133.1 data. The situation is somewhat less favorable, since there are several discrimination failures in this group. The largest failures are circled in each panel; they all correspond to sequences threaded onto the same model structures: 1JLT, 1AOK, and 1PP2. Thus, all the discrimination failures are produced by purple or grey points. 1PP2 also has a negative *S*_thread _when it is threaded onto the 1JLT model. Excluding these points, the variation of *S*_thread _is rather smooth in the 1FX9, 1PP2, and 1JLT panels. In the other panels, the variation of *S*_thread _is less regular, although large Irmsd values do correspond to large or intermediate energies. All these other panels correspond to weak complexes (see the association energies and buried surface areas in each panel's header). Thus, some or all of the corresponding, experimental binding modes could be artefacts of the crystal environment; this would explain the observed violations of the energy/structure correlation. For this ISG, we compared the sequence identity at the interface to the overall sequence identity for several pairs of complexes that share the same interaction mode. In 7 cases out of 7, the overall sequence identity was higher than the interface sequence identity.

Overall, the procedure is much more successful than suggested by the simplistic analysis shown in Fig. 2. The energy/structure correlation is mainly violated by a few models, corresponding to one or a small group of very similar binding modes. In both ISGs analyzed, some or all of these violations may be due to experimental binding modes induced artificially by the crystal environment. Even if the violations are pure artefacts of our modelling procedure, their number is small enough so that a more sophisticated modelling procedure could be used for their further analysis. Indeed, a larger number of high-Irmsd models are clearly eliminated by our energy values. A clustering of binding modes and their detailed energy analysis was needed to reveal this more promising picture. Such an analysis had not been performed in the past.

## Discussion and conclusion

For individual proteins, structure-based homology modelling is by far the most important prediction method. Most of the difficulties that exist for individual protein modelling can also affect complex modelling. However, complexes include an additional structural level, namely quaternary structure. Therefore additional difficulties occur, especially for large, multi-domain, multi-protein complexes. Here, we limited ourselves to the simplest class of complexes: binary complexes between single domain proteins. These already present several specific difficulties, at least four of which were illustrated in this work. First, in the PDB, there are far fewer structures of complexes than of individual proteins. Second, it is often hard to distinguish biologically-meaningful complexes from those induced by the crystal environment. Third, two proteins with similar structures may have very different modes of interaction with a third protein, simply because a few surface residues differ. Fourth, domains within the same protein may shift with respect to each other in different environments.

To overcome these difficulties, we took two main steps. We limited ourselves to binary complexes between single-domain proteins, and we identified complexes that have a weak association free energy (*E*_*int *_>-10 kcal/mol). When comparing two complexes, we also paid care to the definition of equivalent residues (see Methods), so that a simple and intuitive measure of structural distances could be used (the Irmsd), and to the treatment of gaps in the threading procedure.

We obtained two main results. First, complexes that share over 35% sequence identity usually share similar structures and interaction modes. The same qualitative result was obtained in earlier studies [24]. Here, however, our dataset is simpler, our analysis somewhat more detailed, and the overall result is more clearcut. Note that the reverse situation has also been observed: complexes with weak sequence homology can occasionally have similar binding modes. The literature provides just a few known cases, such as cytochome c' and its homolog, the Erv2p thiol oxydase [41]. The precise value of the sequence identity threshold obtained here, 35%, obviously depends on the details of our data set and our alignment method. Exceptions to the similarity principle were either weakly-stable complexes or a few unusual cases (such as domain swapped structures). Below 35% identity it is common to find different interaction modes for homologous complexes.

Our second main result is the imperfect but still respectable success rate obtained when comparing near-native and non-native models (Figs. 3 and 4). The energy/structure correlation is mainly violated by a few models, corresponding to one or a small group of similar binding modes. In both ISGs analyzed above, some of the failures correspond to weakly-stable complexes. These failures and possibly others may be due to experimental binding modes induced artificially by the crystal environment. In general, we expect poor discrimination for highly-transient and weakly-stable complexes, which were shown above to depart from the simple similarity principle. We expect that the small number of other failures could be resolved using a more sophisticated, all-atom energy function. Such an energy function will require a detailed model for the protein sidechains (in contrast to DFIRE_*β*_, which only uses the C_*β*_); this could introduce additional errors. Note that the performance of DFIRE_*β *_is already comparable to several all-atom energy functions [42,43].

Overall, our results suggest that the simple modelling procedure applied here could help identify and characterize, at least in a preliminary way, protein-protein complexes. The next step is to apply it on a genomic scale [17].

## Methods

We describe first the procedure used for the structural and sequence comparisons. Next, we describe the energy function employed. Finally, we describe the structure modeling procedure, which involves a simple threading technique.

### Measuring similarities between pairs of protein domains

The dimers within each ISG were compared in terms of their structures and sequences. To compare two complexes, A_*i*_:B_*i *_and A_*j*_:B_*j*_, we first superimpose the structures of A_*i *_onto A_*j *_and B_*i *_onto B_*j*_. Both pairs were aligned using the MATRAS web server [44], which provides a local structural alignment. MATRAS uses a heuristic method to solve the problem of the structural alignment of two proteins. It employs different similarity scores which are all constructed by applying a Markovian Transition Model to observed data frequencies [45]. A first, rough alignment of the two proteins is obtained by superimposing pairs of secondary structures. This initial alignment is refined by performing two consecutive dynamic programming alignments. Each one uses a specific score and an affine gap penalty. The first score is an "environment score", characterizing the local chain structure combined with a binary description of the solvent accessibility [45]. The second dynamic programming alignment uses a "distance score" between pairs of residues in each protein. This score function was parameterized based on the observed pairwise distances between C_*β*_'s in proteins.

**Figure 5:**
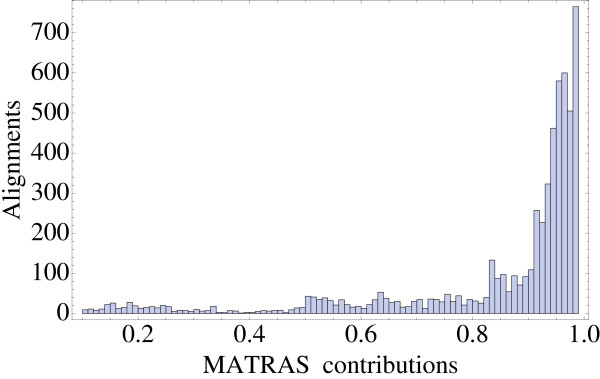
**MATRAS contributions to the alignments**. Length of the MATRAS structural alignment, as a fraction of the total alignment length (not counting gaps). For 9407 out of 19260 alignments, the structural alignment covered 100% of the sequence; these cases are not represented.

The final alignments usually included only a subset of one or both proteins (8054 out of 9630 pairs of dimers, corresponding to 11055 out of 19260 alignments). The regions flanking the aligned segments were sometimes quite large; for 6.9% of the alignments, they represented over 20% of the sequence length (Fig. 5). In these cases, we supplemented the MATRAS alignment with a sequence-based alignment of the regions not aligned by MATRAS. Indeed, we want to characterize the relation between sequence/structure similarity for single-domain, binary complexes. While it is reasonable to limit ourselves to regions with a significant sequence homology, there is no reason to exclude regions where the structures are less similar (i.e., they are not aligned by MATRAS). If we excluded such regions, we would assume, not prove a correlation between sequence and structure similarity. It is more appropriate to take into account flanking regions whose sequences can be aligned, even though their structures were not aligned by MATRAS.

Therefore, we aligned separately the sequences N-terminal and C-terminal with respect to the segment aligned by MATRAS. We used the EMBOSS implementation [46] of the Needleman and Wunsch algorithm [47], with 10 and 0.5 as penalties for the opening and the extension of gaps. This way, we could include additional N- or C-terminal regions in the final alignment. The two alignments (with and without the flanking regions) define two sets of equivalent residues in A_*i *_and A_*j *_(respectively, B_*i *_and B_*j*_).

The equivalent residue sets were then used to compute structural deviations, as follows. The largest of the two domains A_*i *_and B_*i *_was chosen, say A_*i*_. It was superimposed onto A_*j *_using the *C*_*α *_coordinates of equivalent residues. Then, an rms coordinate deviation was computed for B_*i *_and B_*j*_. This deviation measures the displacement of the smaller domain, B_*j*_, at the surface of the larger, A_*j*_, when going from the A_*i*_:B_*i *_complex to A_*j*_:B_*j*_. The rms deviation is computed for all equivalent residues, defined by the larger alignment (structural alignment, plus sequence alignment of flanking residues). We refer to this deviation measure as an "interaction rmsd", or Irmsd [48]. Occasionally, we also employ a deviation computed for interface residues only.

### Measuring the stabilities of the complexes

An energy function is used to compare the different complexes. We rely on pairwise statistical potentials derived in previous studies [40,43]. The stability of a given complex can be characterized by the total energy of interaction between the partners. Alternate structures will be constructed for each dimer using as templates other, homologous dimers from the same ISG. Therefore, a method is also needed to thread the sequence of a given complex onto the structure of another, homologous complex.

#### Statistical energy function for protein-protein interactions

For a complex A:B, an energy score *E*_*int *_is defined:

(1) $$E_{int}(C,S,U)=\sum_{l,m}C_{lm}U(S_{l},S_{m}).$$

Here, *C *is an atomic or residue contact map describing the A:B interface; *l*, *m *are amino acid positions in A and B, respectively; *S*_*l*_, *S*_*m *_are the corresponding amino acid types; and *U *is a table of interaction parameters. This score is computed using either of two knowledge-based energy functions. The first is a residue-based energy function described earlier [40]. It uses a residue-residue contact map. If two nonhydrogen atoms from two residues belonging to the two proteins are less than 4.5 Å apart, the residues are said to be in contact. The interaction parameters form a 6 × 6 table, corresponding to six groups of amino acids: {LVIMCAGSTPFYW}, {ASTP}, {FYW}, {EDNQ}, {KRH}, and {G}. This very simple energy function performed well in several applications [40]. The second energy function is atom-based. It is taken from the DFIRE potentials developed by Zhou and coworkers [42,43]; the protein structures are reduced to their backbone and *C*_*β *_atoms. We refer to it as the DFIRE_*β *_potential.

#### Threading a sequence onto a structure

Each experimental, or "native" complex is compared to a series of modelled complexes. Modelling is done by threading the sequence of the native complex, A_*i*_:B_*i*_, onto the structures of homologous complexes A_*j*_:B_*j*_. Hence, the target dimer A_*i*_:B_*i *_and the template structure A_*j*_:B_*j *_are always part of the same ISG. An interaction energy is computed for each modelled complex. Ideally, templates that are structurally close to the native complex should lead to models with strong interaction energies. Templates that are structurally more distant should lead to poorer energies.

Mapping the A_*i*_:B_*i *_sequence onto the A_*j*_:B_*j *_template structure is a classic threading problem [49-52]. Here, threading is performed very simply, by changing each amino acid type in A_*j*_:B_*j *_to the type found at the equivalent position in A_*i*_:B_*i*_. Equivalency of positions is defined by the alignment described above. Gaps are handled as follows. When a residue *R *of the template A_*j*_:B_*j *_is aligned with a gap, we add a distance-dependent energy penalty *P *to its other interactions. By trial and error, we found that the following form worked well:

(2) $$P=\alpha \sum_{k,l}\frac{A}{D_{k,l}^{2}},$$

where the sum is over the atoms *k *of the "gapped" residue *R *and all the atoms *l *of the target protein. *A *is a real number, empirically set to 10 kcal/mol and *α *is a dimensionless DFIRE scaling factor, *α *= 0.0157 [43]. With this form, the penalty *P *is roughly equivalent to applying a 4.6 kcal/mol cost for removing a residue from the rim of a protein-protein interface [53]. No penalty is applied for the opening or extension of gaps at the beginning or the end of either polypeptide chain (A_*j *_or B_*j*_). Indeed, the definitions of domain boundaries within SCOP are somewhat imprecise, so that differences at the beginning or the end of a domain should not be penalized.

For each threading of a pair of sequences A_*i*_:B_*i *_onto a template complex A_*j*_:B_*j*_, a score *S*_thread _is computed, based on the protein-protein interaction energy:

(3) *S*_thread _= *E*_*int*_(*s*_*i*_, *C*_*j*_) - *E*_*int*_(*s*_*i*_, *C*_*i*_).

Here, *E*_*int*_(*s*_*i*_, *C*_*j*_) is the interaction energy of the A_*i*_:B_*i *_sequence, *s*_*i*_, in its threaded conformation, *C*_*j *_(i.e., threaded onto the A_*j*_:B_*j *_structure). *E*_*int*_(*s*_*i*_, *C*_*i*_) is the energy of the *s*_*i *_sequence in its own, native conformation, *C*_*i*_. If the sequence *s*_*i *_is threaded onto its own, native structure, the score is zero. If the sequence *s*_*i *_is poorly suited to the threaded conformation *C*_*j*_, the score is positive. Occasionally, one can obtain a negative score, in which case the threaded conformation may represent a superior mode of interaction for the A_*i*_, B_*i *_proteins. This might occur if the "native" A_*i*_:B_*i *_structure is, in fact, an artefact of the crystal environment.

#### Basic criteria detecting crystal interfaces

By inspecting all the complexes with PQS [31] and applying a threshold of ten for the minimal number of contacts in a complex (see above), we eliminated many spurious interactions from our dataset. However, large and physically-plausible interfaces that are, in fact, crystal contacts remain in the dataset. This is actually useful, since they provide alternate models that can be compared to even larger, biological interfaces. Following previous studies [29,30], three main indicators are used above to differentiate crystal and biological interfaces: the DFIRE_*β *_interaction energy *E*_*int*_, the number of interfacial contacts, and the total surface buried upon dimerization.

## Authors' contributions

GL: Performed calculations, analyzed data, wrote paper. TS: Designed research, analyzed data, wrote paper.
